# Effects of total abdominal irradiation on gut microbiota and metabolome during acute tissue injury

**DOI:** 10.3389/fmicb.2025.1702694

**Published:** 2025-12-05

**Authors:** Lina Lu, Qingyu Huang, Chao Sun, Shuhe Kang, Pen Jin, Xin Wang, Xingping Luo, Jia Li

**Affiliations:** 1 Key Laboratory of Environment-Friendly Composite Materials of the State Ethnic Affairs Commission, Gansu Provincial Biomass Function Composites Engineering Research Center, Key Laboratory for Utility of Environment-Friendly Composite Materials and Biomass in University of Gansu Province, Gansu Province Research Center for Basic Sciences of Surface and Interface Chemistry, College of Chemical Engineering, Northwest Minzu University, Lanzhou, Gansu, China; 2 Institute of Modern Physics, Chinese Academy of Sciences, Lanzhou, Gansu, China

**Keywords:** radiation-induced intestinal injury, acute phase of injury, gut microbiota, metabolome, TLR4/MYD88/NF-κB signaling pathway

## Abstract

**Objective:**

Radiation-induced intestinal injury is the most common complication following radiotherapy for pelvic tumors. Effective clinical treatments remain limited, and its underlying mechanism remains unclear. Using a mouse model, this study dynamically characterizes the progression of acute radiation-induced intestinal injury through integrated analysis of the gut microbiota and metabolome, thereby supporting the development of rational therapeutic strategies.

**Methods:**

Mice received a single 12 Gy dose of total abdominal irradiation. Feces were collected for microbiota and metabolomic analysis, and intestinal tissues were harvested at 24 h and 3 days post-irradiation. These tissues underwent both histopathological assessment and analysis of inflammatory signaling pathways.

**Results:**

Total abdominal irradiation induced severe intestinal injury. At 24 h post-irradiation, intestinal mucosal cell nuclei were fragmented and intestinal permeability increased. The damage progressively worsened, and by 3 days, villi had shortened, nuclear fragmentation was more extensive, and eosinophilic granulocytes had infiltrated the tissue. Bioinformatic analysis of microbiota data revealed gut dysbiosis during the acute injury phase, characterized by reduced *α*-diversity, an elevated abundance of *g_Escherichia-Shigella, f_Enterobacteriaceae*, and decreased levels of *f_Ruminococcaceae*, *g_Lachnospiraceae_NK4A136*, and other butyrate-producing bacteria. This dysbiosis led to elevated fecal lipopolysaccharide levels and activation of the TLR4/MyD88/NF-κB inflammatory signaling cascade. Kyoto Encyclopedia of Genes and Genomes (KEGG) pathway enrichment analysis indicated that abdominal irradiation predominantly affected lysine degradation, arginine and proline metabolism, primary bile acid synthesis, tryptophan metabolism, taurine metabolism, and sphingolipid metabolism. The effects on lysine degradation, sphingolipid metabolism, and primary bile acid biosynthesis were especially pronounced.

**Conclusion:**

Overall, these data indicate that radiation exposure disrupts both the gut microbiota and metabolome during the acute injury phase, reducing beneficial bacteria such as *f_Ruminococcaceae* and *Bifidobacterium* while promoting the proliferation of harmful bacteria such as *g_Escherichia-Shigella*, which in turn triggers an inflammatory metabolic cascade. Early restoration of a normal gut microbiota could be one of the potential steps to mitigate the radiation effect based on prior literature. These findings provide a scientific basis for future research into microbiota- and metabolome-targeted therapies aimed at mitigating radiation-induced intestinal toxicity.

## 1 Introduction

Malignant tumors have constituted the primary global disease burden since the early 21st century, with incidence and prevalence continuing to rise steadily. International multicenter surveillance data report more than 52,900 new confirmed cases and over 27,000 cancer-related deaths daily (Liu B. et al., 2024). According to the 2022 report by the World Health Organization’s International Agency for Research on Cancer (IARC), new cancer cases worldwide have surpassed 20 million and are projected to reach 35 million by 2050 (Bray et al., 2024; Siegel et al., 2025). Radiotherapy serves as a cornerstone of comprehensive treatment for solid tumors, playing an essential role in controlling local lesions, alleviating symptoms, and improving survival (Hessels et al., 2022; Wang X. et al., 2024). Nevertheless, intestinal tissue is highly sensitive to ionizing radiation, rendering radiation-induced intestinal injury (RIII) one of the most frequent clinical manifestations of acute radiation syndrome (Jingjing et al., 2025). Epidemiological studies suggest that nearly 90% of patients receiving abdominopelvic irradiation develop varying degrees of gastrointestinal symptoms within weeks after starting treatment (Wang K. et al., 2024). Without timely repair, acute injuries may progress into irreversible late complications that substantially impair patients’ quality of life and restrict radiotherapy dose escalation, thereby limiting further improvement in therapeutic efficacy (Natuhwera et al., 2025). Unfortunately, current clinical interventions remain largely symptomatic, and the U. S. Food and Drug Administration (FDA) has not yet approved any specific prophylactic or therapeutic agents for RIII (Li Q. et al., 2025). This is largely due to the absence of a systematic and in-depth understanding of the injury mechanisms and progression during the acute phase.

Recent advances in metagenomics, metabolomics, and multi-omics integration have extensively elucidated the crucial role of gut microbiota and their metabolites in preserving intestinal barrier integrity and systemic immune homeostasis (Mitra et al., 2025; Li et al., 2024). Similar integrative approaches have revealed spatial and temporal dynamics of gut microbial development and functional specialization in different intestinal regions (Ma et al., 2024). Consequently, targeted modulation of microbiota-host interactions has become a promising strategy for preventing and treating RIII (Lu et al., 2024; Chen et al., 2024). For instance, dietary omega-3 polyunsaturated fatty acids have been shown to beneficially modulate gut microbiota composition and metabolic outputs in animal models (Yang et al., 2025). Traditional herbal agents such as Astragalus have also been shown to enhance intestinal barrier integrity and immune function by regulating gut microbial composition, supporting the potential of microbiota-centered therapeutics (Su et al., 2023). However, systematic evidence is still lacking on how gut microbiota and their metabolic networks respond to irradiation during the acute phase, and how these responses subsequently exacerbate or mitigate host injury.

Therefore, a model of radiation-induced intestinal injury was established in this study. Focusing on two acute-phase time points—24 h and 3 days after total abdominal irradiation (TAI)—the dynamic progression of RIII across four dimensions was systematically analyzed. The four dimensions are the structural and functional damage to the intestinal epithelium, temporal disruptions in gut microbiota composition, metabolomic profiles and the activation level of the TLR4/MyD88/NF-κB inflammatory signaling pathway. The aim is to clarify the mechanism of the “intestinal tissue injury-microbiota-metabolism-immunity” quadruple regulation and the key disturbances in the microbiota-host co-metabolic pathways in acute RIII, providing a theoretical basis and practical targets for the development of precise treatment strategies based on microecological intervention.

## 2 Materials and methods

### 2.1 Experimental animals

Female C57BL/6 mice aged 4–6 weeks were purchased from the Lanzhou Veterinary Research Institute, Chinese Academy of Agricultural Sciences (Lanzhou, China). Animals were housed at 22 ± 2 °C under 12-h light/dark cycles with ad libitum access to standard rodent chow and tap water. After a one-week acclimatization period, all experiments were initiated. All animal procedures complied with institutional guidelines for laboratory animal management and use. The experimental protocol was approved by the Animal Experiment Ethics Committee of Northwest Minzu University and conducted in accordance with its guidelines.

### 2.2 Study design

Eighteen female C57BL/6 mice aged 4–6 weeks were fed standard chow with free access to food and water (Goudarzi et al., 2016; Zeng et al., 2022). After 7 days of acclimatization, the mice were randomly divided into three weight-matched groups: Normal Control Group (Group C), 24-h post-Abdominal Irradiation group (Group M_24h_), and 3-day post-Abdominal Irradiation group (Group M_3d_). Except for Group C, animals in the M groups received abdominal X-ray irradiation (12 Gy) at Gansu Provincial Academic Institute for Medical Research to establish the abdominal radiation-induced intestinal injury model. The source-to-skin distance was 100 cm with a dose rate of 2 Gy/min. Animals were attentively cared for until full recovery from anesthesia after irradiation. Fecal samples from each group were aseptically collected at 24 h and 3 days post-irradiation (PI), aliquoted into pre-sterilized cryovials, and stored at −80 °C.

### 2.3 Histopathological analysis

For histological analysis, intestinal tissue specimens were fixed in paraformaldehyde buffer at room temperature for 24 h, dehydrated through a graded ethanol series, and embedded in paraffin. Tissue sections of 5 μm thickness were prepared for hematoxylin and eosin staining (HE) and terminal deoxynucleotidyl transferase dUTP nick end labeling staining (TUNEL). Subsequent results were analyzed using digital image scanning and CaseViewer software.

### 2.4 Immunohistochemical analysis

Intestinal tissue paraffin sections of 4 μm thickness were prepared. Deparaffinized sections were rehydrated, sections were incubated with specific antibodies against tight junction proteins zonula occludens-1 (ZO-1), Occludin, and Claudin-1, followed by staining with corresponding secondary antibodies. Horseradish peroxidase complexes were detected using 3,3′-diaminobenzidine as the substrate. All sections were analyzed with six fields of view (200×) measured per group. When taking screenshots, the entire field of view was filled with tissue so that the background light of each image was consistent. Image-Pro Plus 6.0 software was used to select the same brown-yellow color as the unified standard for judging all positive images; the positive integrated optical density (IOD) and pixel area (AREA) of the tissue were obtained by analyzing each image. The average optical density (AO) was obtained by the formula: AO = IOD/AREA, and larger AO values indicate greater positive-expression levels.

### 2.5 Gut microbiota analysis

DNA extraction and 16S rRNA gene amplification were performed following methods described previously (Liu Y. et al., 2024; Wang et al., 2025). Fecal samples were thawed on ice, and total genomic DNA was extracted with the PowerSoil DNA Isolation Kit (MoBio Laboratories, Carlsbad, CA, USA), according to the manufacturer’s instructions. The 16S rRNA gene V3-V4 region specifific primers are 341F (CCTAYGGGRBGCASCAG) and 806R GGACTACNNGGGTATCTAAT. The 16S rRNA gene was analyzed to evaluate bacterial diversity using the Illumina Hiseq platform (Novogene Co., Ltd., Beijing, China).

### 2.6 Gut metabolites and short-chain fatty acids detection

Gut metabolites detection method: 100 mg fecal samples were added to pre-labeled 2 mL centrifuge tubes, followed by addition of 300 μL purified water and one pre-chilled steel bead. Samples were vortexed for 5 min and incubated on dry ice for 10 min. After removing the steel bead, 500 μL pure methanol (containing 1 ppm 2-chloro-L-phenylalanine) was added. Samples were vortexed at 2500 rpm for 5 min, then incubated on dry ice for 10 min. After centrifuging the samples at 4 °C for 10 min at 12,000 rpm, 600 μL of the supernatant was transferred to a new 1.5 mL centrifuge tube and placed in the concentrator for concentration. The dry residue was added with 100 μL of 5% methanol, and then vortexed at 2500 rpm for 5 min. The samples were then centrifuged at 12,000 rpm and 4 °C for 10 min. The supernatant was taken and placed in the sample vial for LC–MS analysis.

Characteristic peaks were identified and quantified using Mass Profiler Professional software (Agilent), which provided mass-to-charge ratios, retention times, and other relevant data. Data collection was performed with an UHPLC (Agilent: 1290 Infinity LC), equipped with a Waters T3 C18 chromatographic column (100 mm × 2.1 mm, 1.8 μm). Gradient elution was performed in both positive and negative ion modes with the column temperature maintained at 35 °C and a flow rate of 0.3 mL/min. The mobile phases consisted of 0.01% formic acid in water (Phase A) and pure acetonitrile (Phase B). Each sample was analyzed once in positive ion mode and once in negative ion mode using a high-resolution quadrupole time-of-flight mass spectrometer (QTOF/MS-6545, Agilent). The electrospray ionization (ESI) source operated at 250 V for positive ion mode and 1,500 V for negative ion mode, with the gas temperature set to 325 °C.

Short-chain fatty acids (SCFAs) detection method: the concentration of SCFAs in feces was determined by the chloroformate propyl ester derivatization method and gas chromatography–mass spectrometry (GC–MS). In brief, fecal samples (50–150 mg) were placed in 1000 μL of 0.005 mol/L sodium hydroxide aqueous solution containing internal standard (5 μg/mL butyric acid) and mixed and centrifuged. Five hundred microliters of the supernatant was transferred to a 10 mL disposable glass centrifuge tube, then 300 μL of water, 500 μL of PrOH/Py mixed solvent (3,2, v/v) and 100 μL of chloroformic acid propyl ester were added, and the mixture was vortexed. Then, the Agilent 7890A gas chromatography system and Agilent 5975C mass spectrometer detector were used for detection.

### 2.7 Enzyme-linked immunosorbent assay

Levels of the inflammatory mediators tumor necrosis factor *α* (TNF-α) and interleukin (IL)-6 in intestinal tissue and levels of lipopolysaccharides (LPS) in feces were quantified using murine-specific Enzyme-linked Immunosorbent assay (ELISA) kits, according to the manufacturer’s instructions (Shanghai Enzyme-linked Biotechnology Co., Ltd., Shanghai, China).

### 2.8 Western blot analysis

Toll-like receptor 4 (TLR4), myeloid differentiation factor 88 (Myd88), and nuclear factor kappa B (NF-κB) protein expression was examined by western blot. Intestinal tissue samples were first cut into pieces with scissors and then lysed in RIPA buffer (Cell Signaling Technology, Inc) supplemented with protease inhibitors (Roche Applied Science) for 30 min on ice for protein extraction. After protein concentrations were measured by the bicinchoninic acid assay (Beyotime Institute of Biotechnology, Shanghai, China), 50 μg total protein samples were separated via sodium dodecyl sulfate–polyacrylamide gel electrophoresis (10% acrylamide gel) using a Bio-Rad Trans-Blot system (Hercules, CA, USA) and transferred onto a polyvinylidene fluoride membrane, which was then blocked with 5% nonfat milk at room temperature for 1 h. Membranes were incubated with primary antibodies against TLR4 (ab22048; 1:2000), MyD88 (ab199247; 1:2000), NF-κB (ab16502; 1:1000), and *β*-actin (ab8226; 1:2000; all from Abcam, Cambridge, UK) at 4 °C overnight. After washing with Tris-buffered saline with Tween 20 (TBST), membranes were incubated with the appropriate horseradish peroxidase–conjugated secondary antibodies for 1 h at room temperature. Enhanced chemiluminescence western blot detection reagents (GE Healthcare, Piscataway, NJ, USA) and Fluor Chem FC2 software (Alpha Innotech Corporation, San Leandro, CA, USA) were used to visualize results and quantify bands.

### 2.9 Statistical analysis

One-way analysis of variance (Tukey’s test) was used to determine differences in microbiota composition between groups. Statistical analysis was performed using SPSS 19.0 software, with *p* < 0.05 considered statistically significant. Screening of differential gut metabolites initially applied the Benjamini-Hochberg method for correction, from which *q*-values were derived. Multivariate statistical analysis of metabolites was mainly performed through orthogonal partial least squares-discriminant analysis (OPLS-DA). Significant differential metabolites were identified using the criteria of variable importance in projection (VIP) ≥ 1, false discovery rate (FDR) < 0.05, and fold change ≥ 2 or ≤ 0.5. Correlations were calculated by Spearman correlation analyses with 95% confidence intervals.

## 3 Results

### 3.1 TAI caused severe damage to intestinal tissue

**Figure 1:**
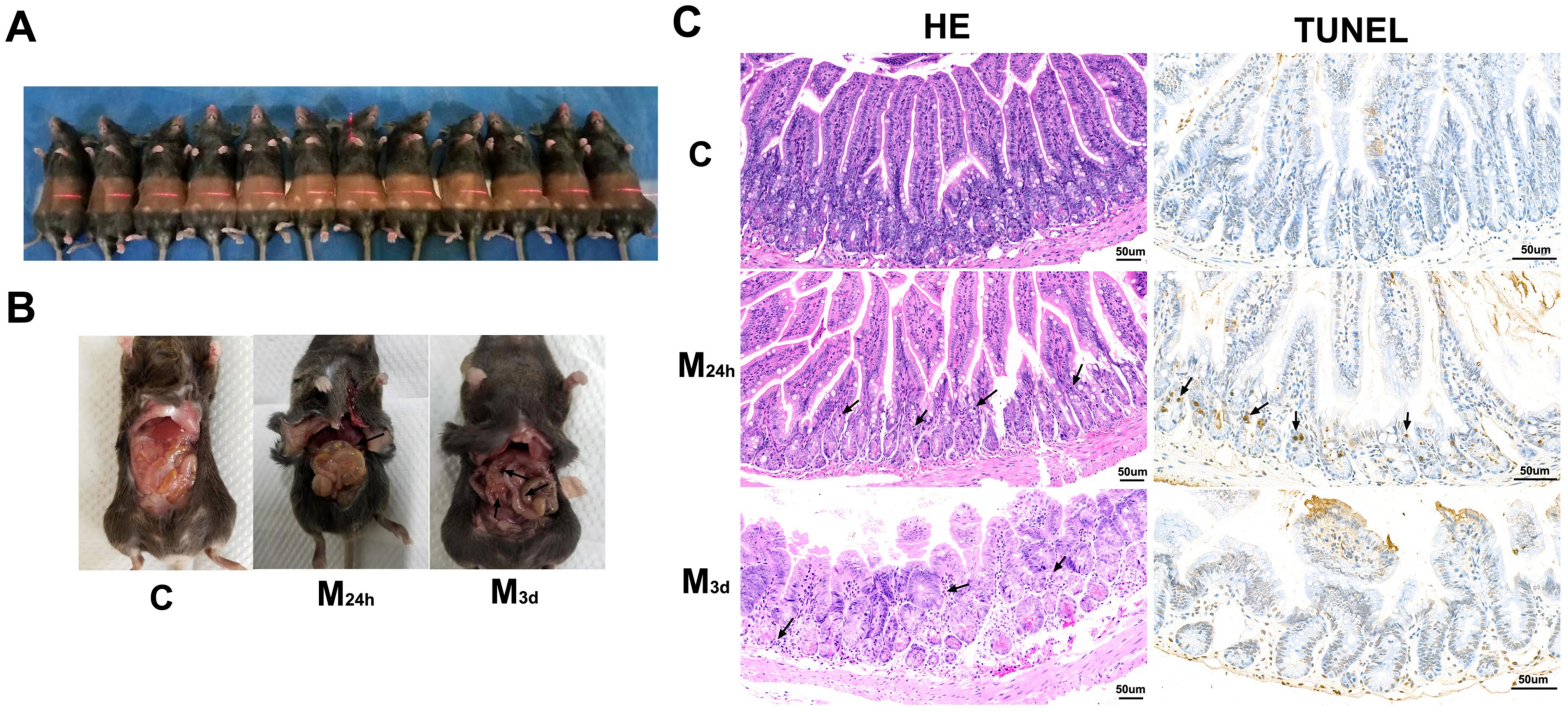
Effects of TAI on histopathological morphology and apoptosis in intestinal tissue. **(A)** After anesthesia, the whole abdominal region of animals was irradiated with X-rays (12 Gy) **(B)** Flatulence induced by TAI **(C)** HE and TUNEL staining (Bars represent 50 μm); C: Control; M_24h_: 24-h post-TAI; M_3d_: 3-day post-TAI.

TAI resulted in severe intestinal damage (Figure 1A). Normal intestinal tissue exhibited a pinkish hue, good elasticity, with no signs of gas accumulation or hemorrhage. At 24 h PI, the intestinal tissue had become dark red and exhibited reduced elasticity. Varying degrees of gas accumulation were visible within the lumen, with severe cases presenting a bead-like appearance. Three days after irradiation, the intestines remained dark red, with persistent gas accumulation at different levels (Figure 1B, black arrow). This phenomenon may be linked to gut microbiota dysbiosis. Damage to the intestinal mucosal barrier increases the relative abundance of gas-producing bacteria, including *Bacteroides*, *Ruminococcus*, and *Roseburia*. These bacteria generate gas through the fermentation of undigested carbohydrates (Mutuyemungu et al., 2023). HE and TUNEL staining revealed that normal intestinal tissue maintained clearly defined mucosal layers, tall and orderly arranged villi, tightly packed intestinal glands, and normal interstitium. Partial apoptosis and necrosis were observed in epithelial cells of intestinal glands within the intestinal mucosa, accompanied by nuclear fragmentation at 24 h PI (Figure 1C, black arrow). Villous structure remained largely unaltered, with no significant inflammatory cell infiltration detected in the interstitium. By day 3 PI, the intestinal villi were markedly shortened and exhibited degenerative edema, appearing disorganized with partial or complete shedding. The intestinal glands displayed structural disarray, and severe edema was evident in both the lamina propria and submucosa. Eosinophil infiltration was observed in the lamina propria (Figure 1C, black arrow), although significant cellular apoptosis was not apparent. This observation aligns with previous findings (Zeng et al., 2022), which indicate that intestinal tissue damage progressively worsens during the first 3 days following irradiation.

### 3.2 TAI increased intestinal permeability

**Figure 2:**
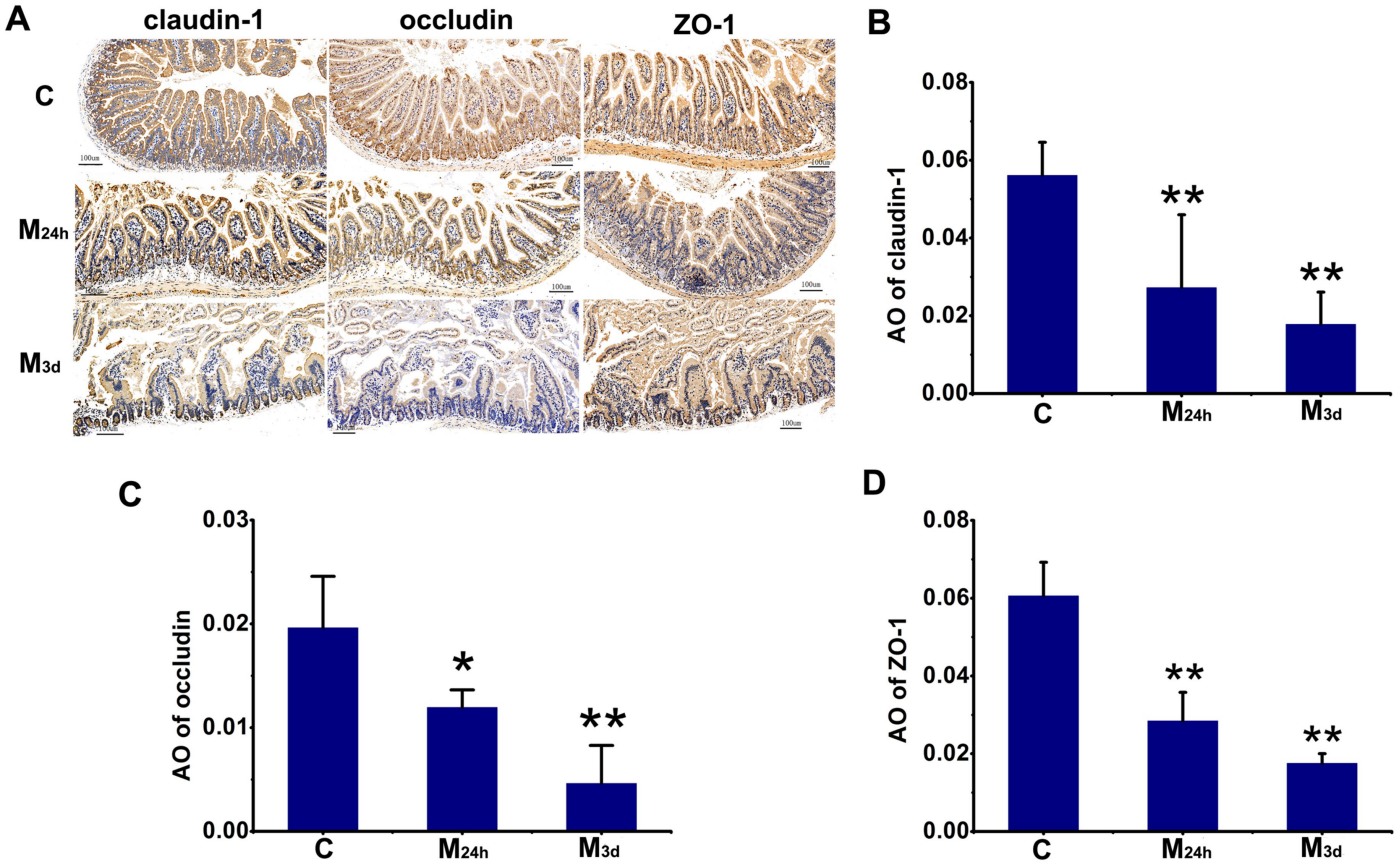
TAI increased intestinal permeability. **(A)** Radiation significantly reduced tight junction protein expression. **(B–D)** Quantitative analysis demonstrated decreased expression of tight junction proteins Claudin-1, Occludin, and ZO-1 post-irradiation (*n* = 6 each); results are presented as mean ± SD; *p < 0.05, **p < 0.01 (Bars represent 100 m; C: Control; M_24h_: 24-h post-TAI; M_3d_: 3-day post-TAI).

TAI reduced intestinal tight junction proteins expression, which increased intestinal permeability. AO measurements showed a dramatic decrease in tight junction protein expression 24 h PI, with a statistically significant difference compared to the control. Expression continued to decline at 3 days PI, demonstrating a highly significant difference from the control (Figure 2). This finding indicates that intestinal barrier function is severely impaired during the acute phase of radiation injury.

### 3.3 TAI led to gut microbiota dysbiosis

**Figure 3:**
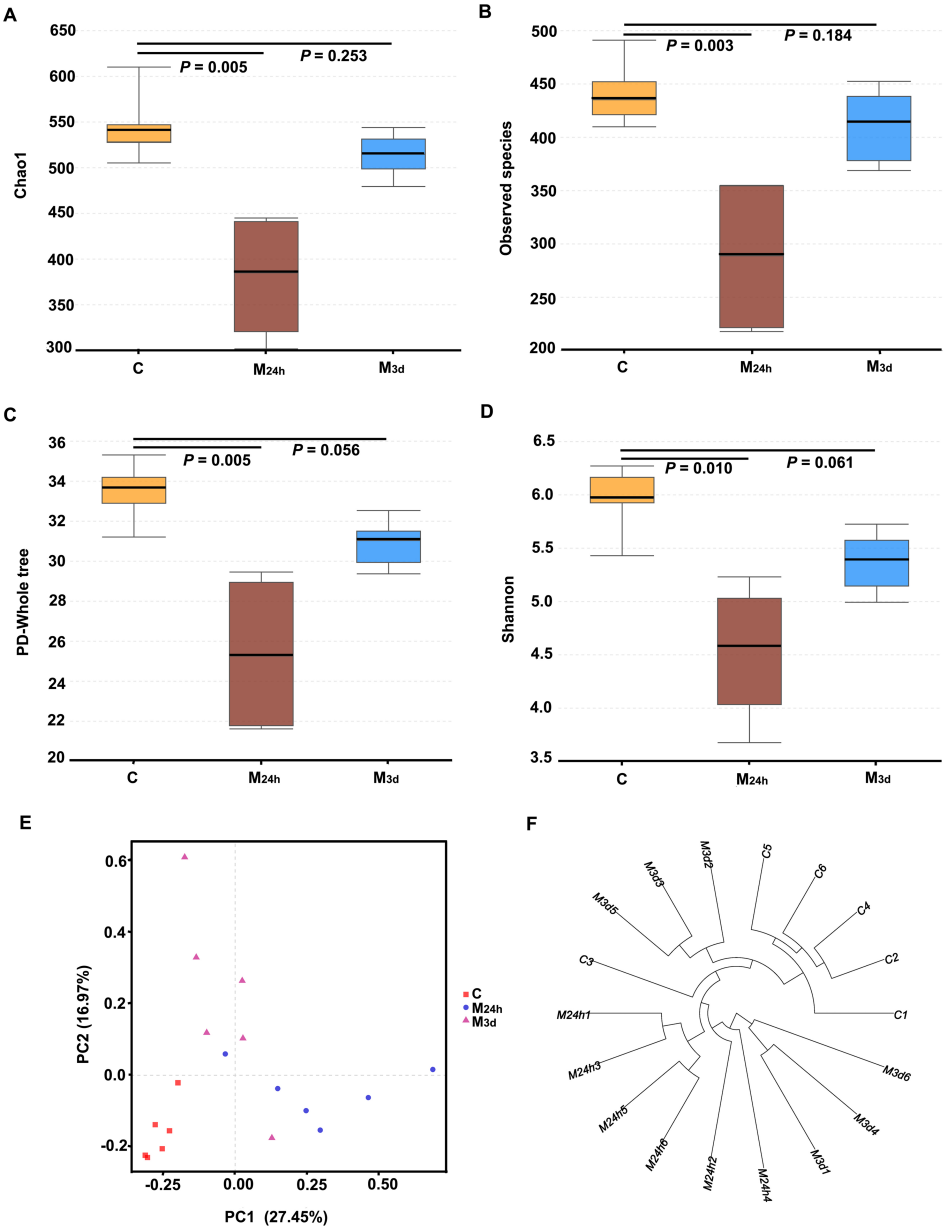
Impact of TAI on gut microbiota diversity and inter-group variations. **(A–D)** Gut microbiota diversity was assessed using Chao1, observed species, PD-whole tree, and Shannon indices (*n* = 6); **(E)** Principal component analysis (PCA); **(F)** Weighted UniFrac cluster analysis; C: Control; M_24h_: 24-h post-TAI; M_3d_: 3-day post-TAI.

The results indicated that TAI reduced gut microbiota diversity, as demonstrated by significant decreases in the Chao1, observed species, PD whole tree, and Shannon indices at 24 h PI compared to the C Group. By 3 days PI, all indices had increased but remained below those of the control group (Figure 3). Principal component analysis (PCA) and cluster analysis revealed clear separation between groups (*p* = 0.001, *R* = 0.506; where *R* > 0 indicates greater inter-group than intra-group variation and *p* < 0.05 denotes statistical significance). Both PCA and weighted UniFrac-based clustering confirmed a pronounced distinction between the normal and irradiated groups. The sustained reduction in *α*-diversity indices, all remaining below normal levels, indicates that TAI induced gut microbiota dysbiosis.

**Figure 4:**
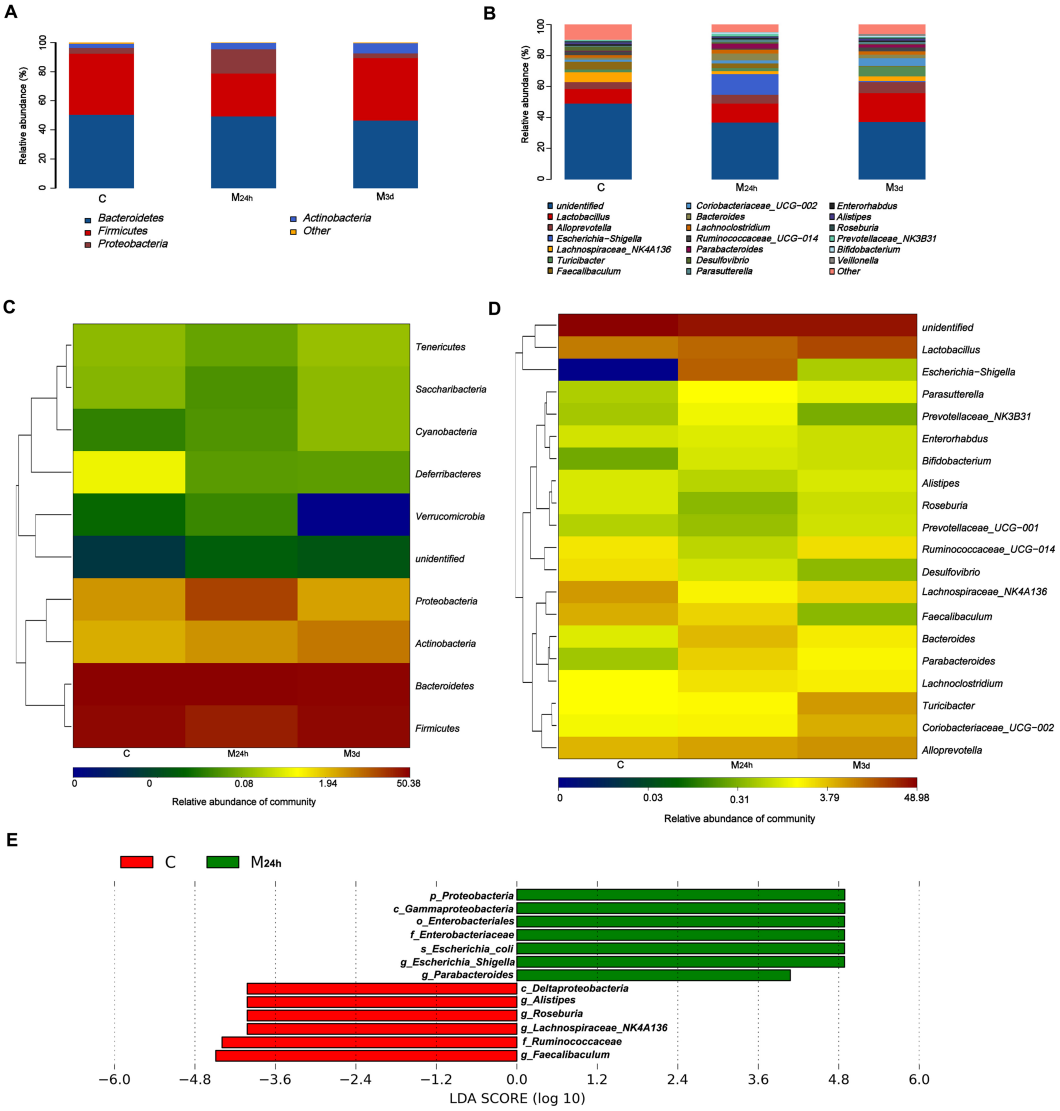
Effects of TAI on gut microbiota composition. **(A,B)** Composition of gut microbiota at the phylum **(A)** and genus **(B)** levels via 16S rRNA gene sequencing; **(C,D)** Differentially abundant bacterial groups based on heatmap at the phylum **(C)** and genus **(D)** levels; **(E)** LEfSe analysis; C: Control; M_24h_: 24-h post-TAI; M_3d_: 3-day post-TAI.

Subsequently, we analyzed changes in gut microbiota composition. At the phylum level, the normal gut microbiota primarily consists of *Bacteroidetes* (approximately 45%), *Firmicutes* (around 40%), *Proteobacteria* (about 5%), and *Actinobacteria* (roughly 5%). By 24 h PI, the relative abundance of *Proteobacteria* had significantly increased by approximately 15–20%, accompanied by a marked reduction in *Firmicutes* (about 15%) and a moderate rise in *Actinobacteria* (around 5%) (Figures 4A,B). Three days after irradiation, the proportion of *Firmicutes* rebounded while *Proteobacteria* declined, though *Actinobacteria* remained elevated by approximately 8%. At the genus level, harmful bacteria represented by *Shigella* increased from undetectable levels to roughly 20% at 24 h PI. Although their abundance decreased by day 3, they remained detectable. Concurrently, the abundance of *Bacteroides* increased and continued to rise through 3 days PI. Multiple beneficial bacteria exhibited reduced abundance 24 h after irradiation, including *Prevotella*, *Lachnospiraceae*, *Roseburia*, *Alistipes*, *Ruminococcus*, and *Faecalibacterium*, among others. Notably, *Faecalibacterium* showed a sustained reduction through 3 days PI (Figures 4C,D). This finding demonstrates that radiation significantly alters the composition of the intestinal microbiota, aligning with previous research (Yi et al., 2023). In the Linear Discriminant Analysis Effect Size (LEfSe) results, biomarkers reflecting the significant impact of TAI on the microbiota were identified. As noted above, these include increased abundance of *Shigella* and *Bacteroides*, and decreased levels of *Prevotella*, *Moraxella*, *Rosebacter*, *Rhodobacter*, *Rumenicola*, and *Enterobacter* (Figure 4E). These significantly altered bacterial communities may influence intestinal metabolism through multiple mechanisms.

### 3.4 TAI induced gut metabolic disorder

**Figure 5:**
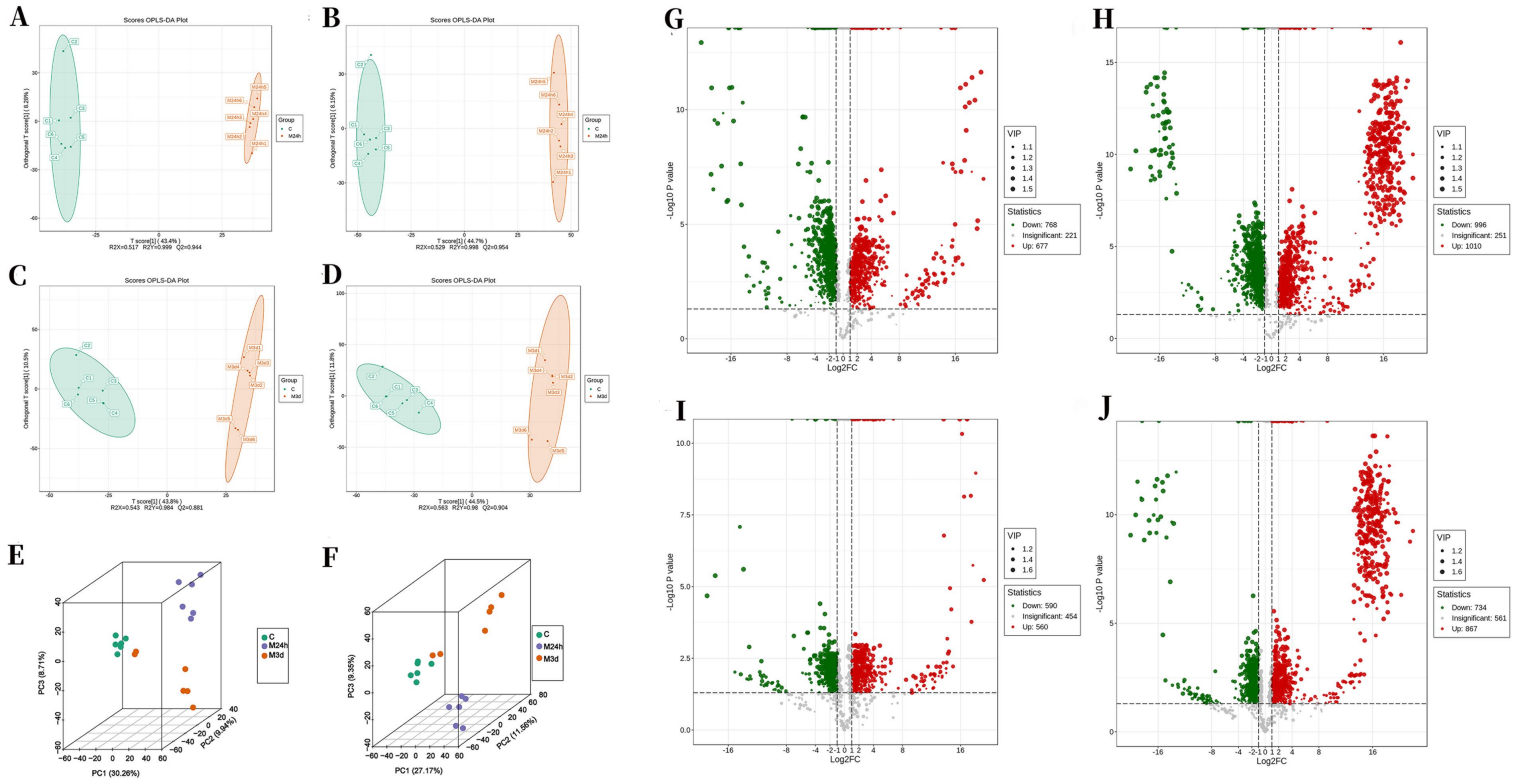
Effect of TAI on gut metabolism. **(A,C,E)** Results from liquid-phase cationic mode measurements; **(B,D,F)** results from liquid-phase anionic mode measurements; **(A,B)** OPLS-DA analysis comparing group C and group M_24h_; **(C,D)** OPLS-DA analysis comparing group C and group M_3d_; **(E,F)** three-dimensional PCA analysis; **(G,I)** Screening of differential metabolites from liquid-phase cationic mode measurements; **(H,J)** Screening of differential metabolites from liquid-phase anionic mode measurements; **(G,H)** comparison between group C and group M_24h_; **(I,J)** comparison between group C and group M_3d_; C: control; M_24h_: 24-h post-TAI; M_3d_: 3-day post-TAI.

This study employed principal component analysis (PCA) to determine the overall differences between the normal control group and the irradiated groups. The three-dimensional analysis of PCA revealed significant inter-group differences between the normal control group and the two irradiated groups (Figure 5). Orthogonal Partial Least Squares-Discriminant Analysis (OPLS-DA) was used to identify the differential metabolites. The results showed that in the positive ion mode of liquid chromatography, the R_2_Y values of the M_24h_ and M_3d_ groups compared with the C group were 0.999 and 0.984 (the closer the value is to 1, the more stable and reliable the model is), and the Q_2_ values were 0.944 and 0.881 (Q₂ > 0.5 indicates an effective model, and Q₂ > 0.9 indicates an excellent model); in the negative ion mode of liquid chromatography, the R_2_Y values of the M_24h_ and M_3d_ groups compared with the C group were 0.998 and 0.98, and the Q_2_ values were 0.954 and 0.904 (Figure 5). These data indicate that gut metabolism is disordered and has significant differences from the normal metabolism during the acute injury period after TAI.

Based on the OPLS-DA results, metabolites differing between groups were preliminarily screened using Variable Importance in Projection (VIP) combined with false discovery rate (FDR) and fold change (selection criteria: VIP ≥ 1, FDR < 0.05, and fold change ≥ 2 or ≤ 0.5). Volcano plot analysis revealed that in the cationic liquid chromatography mode, 768 and 590 metabolites decreased in abundance 24 h and 3 days after irradiation, respectively, while 677 and 560 increased; in the anionic mode, 996 and 734 metabolites decreased at the same time points, whereas 1,010 and 867 increased (Figure 5). These results demonstrate that TAI induces substantial alterations across thousands of metabolites rather than only a few. Radiation-induced intestinal metabolic disorders have also been confirmed in other studies. For instance, a study (Zheng et al., 2021) identified 9 different metabolites at the 7-day time point after TAI, including organic acids and their derivatives, amino acids and their derivatives, etc.

### 3.5 Impact of TAI on differential metabolites and metabolic pathways

**Figure 6:**
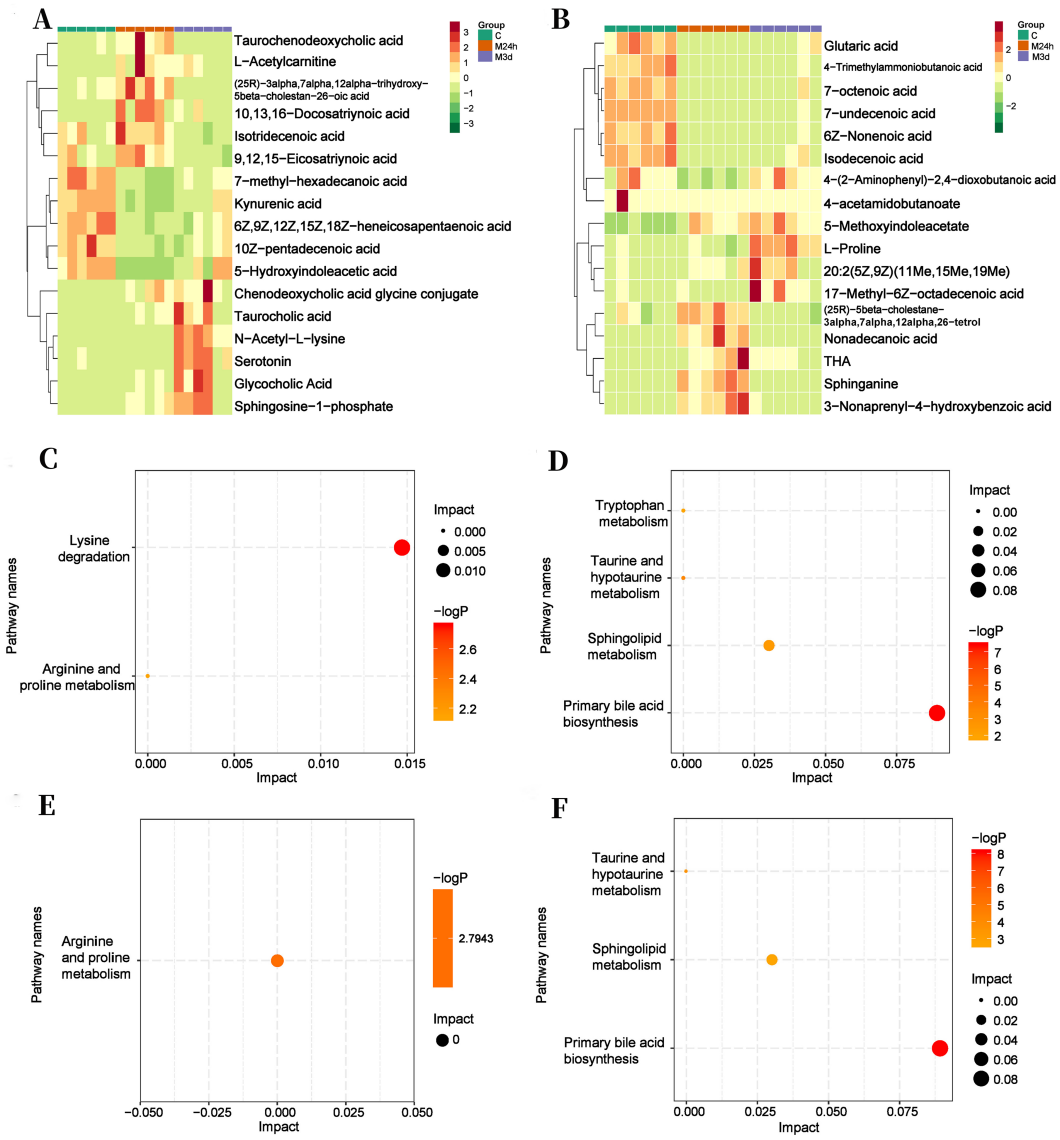
Heatmap illustrating changes in differential metabolite levels and enrichment analysis of KEGG pathways. **(A)** Analysis of results measured in liquid-phase anionic mode; **(B)** Analysis of results measured in liquid-phase cationic mode; **(C,E)** Results measured under liquid-phase cationic state; **(D,F)** Results measured under liquid-phase anionic state; **(C,D)** Comparison between Group C and M_24h_ group; **(E,F)** Comparison between Group C and M_3d_ group; C: Control; M_24h_: 24-h post-TAI; M_3d_: 3-day post-TAI.

To facilitate observation of metabolite variation patterns, significantly differential metabolites underwent normalization and were visualized in a clustered heatmap. Metabolites showing significant changes at both 24 h and 3 days PI compared to the Control were selected. A heatmap was generated based on concentration changes (Figure 6). Substantial alterations occurred in metabolites associated with crucial metabolic pathways under both Liquid-phase Cationic and Anionic states.

The results demonstrated that after irradiation, (i) the key intermediates 4-trimethylammoniobutanoic acid and glutaric acid were both substantially down-regulated in the lysine degradation pathway, with the reduction in glutaric acid being particularly pronounced; (ii) in the primary bile acid biosynthesis pathway, glycocholic acid increased sharply, while the glycine conjugate of chenodeoxycholic acid and taurocholic acid—critical regulators of enterohepatic circulation (Guo et al., 2025)—accumulated progressively over the 3-day PI period, whereas taurochenodeoxycholic acid remained significantly suppressed; (iii) the sphingolipid signaling mediator sphingosine-1-phosphate was markedly up-regulated at both 24 h and 3 days post-exposure; and (iv) 4-acetamidobutanoate, within the arginine–proline metabolic axis, was sharply reduced (Table 1). This finding aligns with previous studies, demonstrating that irradiation induces diverse amino acid metabolic disorders. These disturbances may be associated with the cellular stress response and gut microbiota dysregulation triggered by irradiation (Chen et al., 2022).

**Table 1:** Significantly different metabolites and metabolic pathways.

Compound	Mass	Formula	Group	VIP	FDR	Fold. Change	Type	KEGG Pathway
4-Trimethylammoniobutanoic acid	146.118	C_7_H_16_NO_2_	M_24h_	1.509	0.000	0.098	Down	Lysine degradation
Glutaric acid	132.044	C_5_H_8_O_4_	M_24h_	1.542	1.171E-13	8.111E-07	Down	Lysine degradation
4-acetamidobutanoate	145.073	C_6_H_11_NO_3_	M_24h_	1.321	0	0.062	Down	Arginine and proline metabolism
Glycocholic Acid	465.312	C_26_H_43_NO_6_	M_24h_	1.447	3.527E-06	9.596	Up	Primary bile acid biosynthesis
Taurocholic acid	515.292	C_26_H_45_NO_7_S	M_24h_	1.273	4.312E-08	481823.422	Up	Primary bile acid biosynthesis; Taurine and hypotaurine metabolism
Chenodeoxycholic acid glycine conjugate	449.314	C_26_H_43_NO_5_	M_24h_	1.664	2.925E-10	95764.611	Up	Primary bile acid biosynthesis
Kynurenic acid	189.043	C_10_H_7_NO_3_	M_24h_	1.465	1.238E-06	0.156	Down	Tryptophan metabolism
Sphingosine-1-phosphate	379.249	C_18_H_38_NO_5_P	M_24h_	1.165	0	401.024	Up	Sphingolipid metabolism
5-Hydroxyindoleacetic acid	191.058	C_10_H_9_NO_3_	M_24h_	1.512	0	0.0625	Down	Tryptophan metabolism
4-acetamidobutanoate	145.073	C_6_H_11_NO_3_	M_3d_	1.321	0	0.062	Down	Arginine and proline metabolism
Taurochenodeoxycholic acid	499.297	C_26_H_45_NO_6_S	M_3d_	1.493	2.995E-12	1.829E-06	Down	Primary bile acid biosynthesis
Taurocholic acid	515.292	C_26_H_45_NO_7_S	M_3d_	1.460	5.601E-10	4271664.765	Up	Primary bile acid biosynthesis; Taurine and hypotaurine metabolism
Chenodeoxycholic acid glycine conjugate	449.314	C_26_H_43_NO_5_	M_3d_	1.664	2.925E-10	95764.611	Up	Primary bile acid biosynthesis
5-Methoxyindoleacetate	205.074	C_11_H_11_NO_3_	M_3d_	1.512	0	2.833	Up	Tryptophan metabolism
Nε-Acetyl-L-lysine	188.116	C_8_H_16_N_2_O_3_	M_3d_	1.666	0	16	Up	Lysine degradation
Sphingosine-1-phosphate	379.249	C_18_H_38_NO_5_P	M_3d_	1.373	0	607.950	Up	Sphingolipid metabolism

M_24h_: 24-h post-TAI; M_3d_: 3-day post-TAI.

Enrichment analysis of KEGG pathways indicated that abdominal irradiation predominantly influenced lysine degradation, arginine and proline metabolism, primary bile acid biosynthesis, tryptophan metabolism, taurine and hypotaurine metabolism, and sphingolipid metabolism at 24 h. Lysine metabolism, sphingolipid metabolism, and primary bile acid biosynthesis exhibited the most substantial changes. By 3 days PI, significant alterations were detected mainly in arginine and proline metabolism, primary bile acid biosynthesis, taurine and hypotaurine metabolism, and sphingolipid metabolism, with sphingolipid metabolism and primary bile acid biosynthesis showing particularly pronounced effects (Figure 6). In conclusion, TAI significantly disrupted multiple metabolic pathways in the gut, particularly those governing barrier integrity and inflammatory responses. These changes in metabolic pathways may be associated with radiation-induced intestinal damage and also with the disruption of the gut microbiota after irradiation. These results further emphasize the necessity of targeting these key metabolic pathways for intervention to alleviate radiation-induced intestinal damage.

### 3.6 TAI reduced the levels of short-chain fatty acids

SCFAs are direct metabolites of gut microbiota (Kim et al., 2024), which to a large extent reflects the gut microbiota composition. Building upon the gut microbiota findings, the levels of SCFAs in fecal samples were further quantified. The results showed that the levels of all the detected SCFAs decreased 24 h after irradiation. Among them, the contents of propionic acid, isobutyric acid, isovaleric acid, butyric acid and caproic acid were significantly lower compared to the control group (*p* < 0.01). The contents of SCFAs recovered to some extent, but the contents of propionic acid, isobutyric acid and isovaleric acid were still significantly lower than those of the control group 3 days after irradiation (Table 2). This finding aligns with previous studies (Park et al., 2025), which suggest that a reduction in SCFAs is closely associated with gut microbiota dysbiosis. SCFAs are essential for maintaining intestinal barrier function, regulating immune responses, and promoting overall gut health (Zhu et al., 2025; Lu et al., 2024). A marked decline in their concentration may exacerbate radiation-induced damage to intestinal tissues.

**Table 2:** Effect of TAI on the content of SCFAs.

Items (μg/mL)	C	M_24h_	M_3d_
Propionic acid	3.143 ± 0.773	1.308 ± 0.422**	1.973 ± 0.364**
Isobutyric acid	0.262 ± 0.073	0.051 ± 0.020**	0.136 ± 0.050*
Isovaleric acid	0.209 ± 0.033	0.050 ± 0.020**	0.109 ± 0.047**
Valeric acid	0.146 ± 0.079	0.071 ± 0.049	0.129 ± 0.092
Acetic acid	7.876 ± 3.363	4.648 ± 1.487	6.240 ± 1.972
Butyric acid	1.620 ± 0.373	0.561 ± 0.219**	1.307 ± 0.879
Caproic acid	0.017 ± 0.005	0.010 ± 0.002*	0.015 ± 0.002

Data presented as mean ± SD (*n* = 6). Statistical significance: *p < 0.05, **p < 0.01, compared to the C group (C: Control; M_24h_: 24-h post-TAI; M_3d_: 3-day post-TAI).

### 3.7 TAI increased LPS content and activated inflammatory signaling pathways

**Figure 7:**
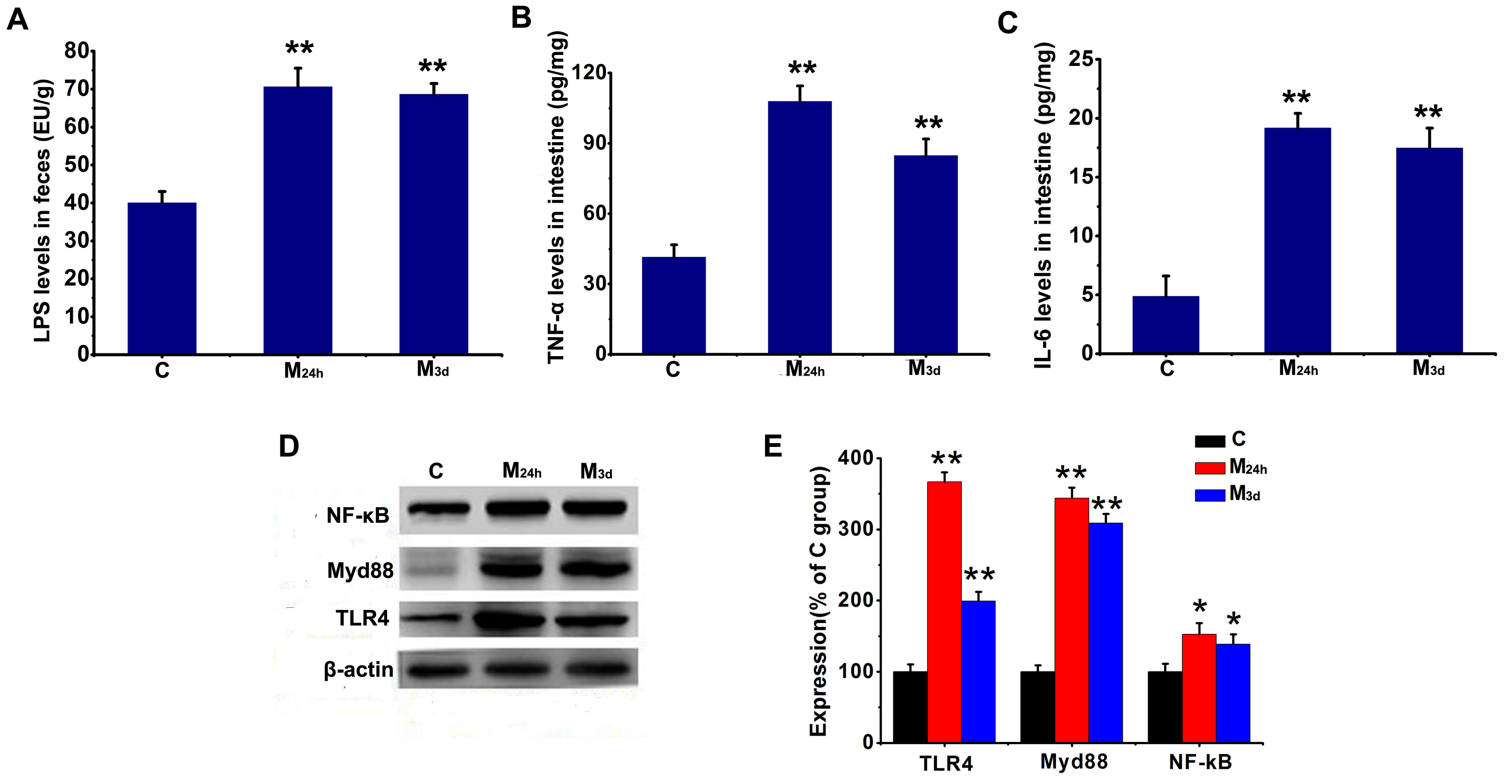
Effects of TAI on LPS, TNF-*α*, IL-6, and the TLR4/Myd88/NF-κB signaling pathway. **(A–C)** Levels of LPS, TNF-α, and IL-6 were measured by ELISA; **(D,E)** levels of TLR4, Myd88, and NF-κB in intestinal tissues were detected by western blotting; data are presented as mean ± SD (*n* = 6). Statistically significant differences: *p < 0.05, **p < 0.01, compared to the C group (C: ontrol; M_24h_: 24-h post-TAI; M_3d_: 3-day post-TAI).

LPS, a component of Gram-negative bacterial cell walls, serves as a critical risk factor for cellular damage. Since TAI substantially alters microbiota composition, we measured fecal LPS levels via ELISA. Results revealed that LPS content in feces increased to approximately 2-fold of the control group at both 24 h and 3 days post-abdominal irradiation, with statistically significant differences (Figure 7A). These suggests that elevated LPS levels resulting from gut microbiota dysbiosis may exacerbate intestinal injury.

TLR4 serves as the principal recognition site for LPS *in vivo*. Binding of LPS to TLR4 activates Myd88, which subsequently triggers NF-κB activation and enhances the release of inflammatory factors such as TNF-*α* and IL-6, thereby exacerbating cellular damage. Based on the LPS findings, the levels of the TLR4/Myd88/NF-κB signaling pathway and inflammatory factors in intestinal tissue were examined. The results showed that TLR4 levels increased nearly 4-fold, Myd88 rose 3-fold, and NF-κB increased by 1.5-fold at 24 h PI compared with the Normal Control Group. TLR4 levels remained elevated by nearly 2-fold, Myd88 sustained a 3-fold increase, and NF-κB persisted at 1.5-fold higher levels at 3 days PI. The inflammatory factor TNF-α increased 2–3 fold at both 24 h and 3 days PI, while IL-6 levels also rose by 3–4 fold (Figure 7). These results indicate that elevated LPS levels resulting from dysbiosis activated the TLR4/Myd88/NF-κB signaling pathway, leading to increased expression of inflammatory factors. This result is consistent with the previous study (Jian et al., 2021), indicating that the increase in LPS content caused by gut microbiota disorder may exacerbate intestinal damage.

### 3.8 Correlation analysis of gut microbiota disorder induced by TAI with intestinal tissue injury and metabolic disorder

**Figure 8:**
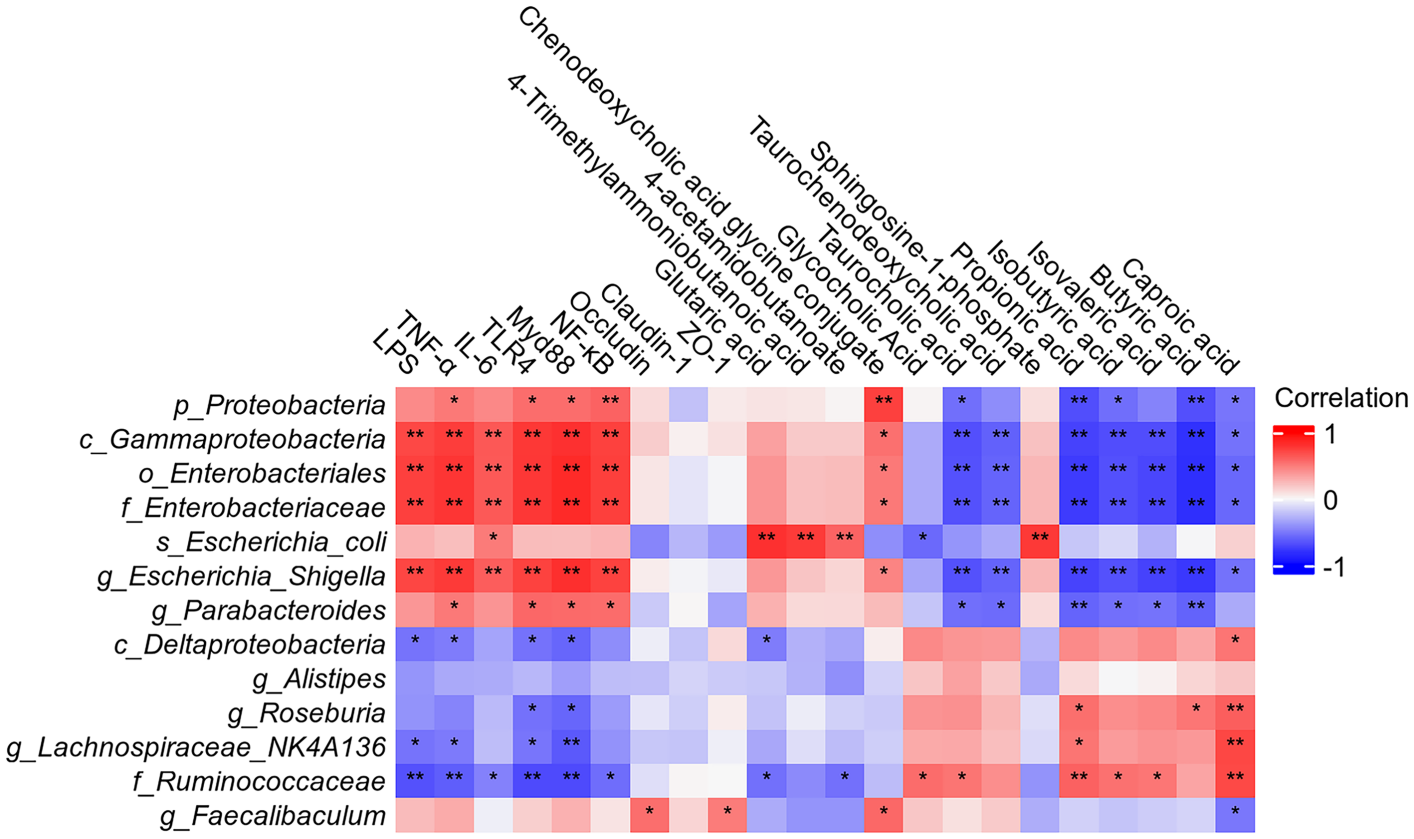
Correlation analysis between the physiological index (LPS, TNF-α, IL-6, TLR4, Myd88, NF-κB, Claudin-1, Occludin, and ZO-1), SCFAs, differential metabolites, and the gut microbiota. A positive correlation was expressed as red and a negative correlation was expressed as blue. The degree of correlation was expressed by intensity of the colors. *p < 0.05; **p < 0.01.

Spearman correlation analysis revealed that the abundances of *g_Roseburia*, *o_Enterobacteriaceae*, and *g_Alistipes* at the genus level, along with *g_Lachnospiraceae*, *f_Ruminococcaceae*, and *Coriobacteriaceae* at the family level, were significantly negatively correlated with intestinal inflammatory markers—including TNF-α, IL-6, TLR4, MyD88, and NF-κB—and significantly positively correlated with gut barrier indicators such as the tight-junction proteins Claudin-1, Occludin, and ZO-1 (Figure 8). Conversely, *Escherichia*–*Shigella* exhibited a significant positive correlation with inflammatory markers and a negative correlation with tight-junction proteins (all *p* < 0.01). The levels of butyric acid and propionic acid correlated positively with *g_Lachnospiraceae* and *f_Ruminococcaceae* (*p* < 0.01) and negatively with *g_Escherichia_Shigella* (*p* < 0.01).

Metabolites linked to primary bile-acid biosynthesis, such as glycochenodeoxycholic acid, showed positive associations with *p_Proteobacteria*, *c_Gammaproteobacteria*, *o_Enterobacteriales*, *f_Enterobacteriaceae*, *g_Escherichia*–*Shigella*, and *g_Faecalibaculum*, whereas taurochenodeoxycholic acid displayed an inverse correlation pattern. Lysine degradation products such as glutaric acid and the arginine/proline metabolite 4-acetamidobutanoate were positively correlated with *s_Escherichia_coli* and negatively correlated with *f_Ruminococcaceae*. Sphingolipid metabolites including sphingosine-1-phosphate also showed a significant positive correlation with *s_Escherichia_coli*.

## 4 Discussion

The intestinal impact of TAI involves a complex series of events, including intestinal tissue damage, gut dysbiosis, metabolic disorders, inflammatory responses, and other contributing factors (Jameus et al., 2024). Two time points PI were selected for the study. The 24-h time point represents the initiation of acute radiation-induced intestinal injury, focusing on early events like DNA damage response and apoptosis initiation, while the 3-day time point marks the start of initial repair (alongside injury progression), characterized by disrupted intestinal barrier and activated regeneration-related genes, with both time points falling within the acute injury phase (Goudarzi et al., 2016; Zeng et al., 2022). A mouse model of abdominal irradiation-induced intestinal injury was established to systematically examine pathological damage in intestinal tissues, gut microbiota alterations, metabolite changes, and relevant signaling pathway expression at 24 h and 3 days PI. These findings clarify the multifaceted effects of abdominal irradiation on the intestine and elucidate their underlying mechanisms.

24 h after 12 Gy TAI, intestinal tissue damage was observed, including nuclear fragmentation in mucosal cells and epithelial apoptosis, accompanied by a marked increase in intestinal permeability. By 3 days PI, the damage had progressed further, exhibiting shortened intestinal villi, eosinophil infiltration, and persistently high intestinal permeability. These observations align with prior studies (Li L. et al., 2025; Parfenov et al., 2025), which indicate that injury to intestinal epithelial cells and disruption of tight junction proteins underlie the increase in intestinal permeability.

The gut microbiota of healthy mammals, including mice and humans, is dominated by the *Firmicutes* and *Bacteroidetes* phyla, while *Proteobacteria*, *Verrucomicrobia*, and *Actinobacteria* remain in relative homeostasis (Hessels et al., 2022; Zhu et al., 2024). Within 24 h after TAI, however, a phylum-level shift occurred: the relative abundance of *Firmicutes* declined sharply, whereas *Proteobacteria* expanded significantly, implying that vacant ecological niches were rapidly colonized by Gram-negative bacteria. Although some restoration of this dysbiosis was observed by day 3 PI, the major phyla did not return to baseline levels, reflecting the microbiota’s limited self-regulatory capacity and delayed recovery kinetics. Further genus-level analysis indicated that TAI induced dysbiosis followed a dual pattern of “opportunistic pathogen expansion and commensal beneficial bacteria suppression.” The potentially pathogenic genus *Shigella*, a Gram-negative facultative anaerobe, surged to approximately 20% relative abundance and remained elevated at day 3. This suggested that genus *Shigella* had a competitive advantage in the post-radiation redox environment. *Bacteroides* also showed sustained elevation after irradiation, likely due to its efficient use of increased mucin oligosaccharides for proliferation. This shift may adversely affect host health through several mechanisms (Wu et al., 2023; Then et al., 2021): *Bacteroides*-derived LPS can activate intestinal immune cells and exacerbate local inflammation, while its overgrowth may disrupt epithelial tight junctions, increase intestinal permeability, and promote endotoxin translocation, thereby amplifying inflammatory responses. Furthermore, *Bacteroides* proliferation may interfere with bile acid metabolism, impairing intestinal function. Notably, excessive *Bacteroides* growth can competitively inhibit beneficial bacteria such as *Prevotella*, *Lachnospiraceae*, and *Ruminococcus* through niche competition, and their decline may further destabilize the microbial community. This imbalance could consequently negatively affect host health. Experimental data confirmed that increased *Bacteroides* abundance coincided with a pronounced reduction in these beneficial taxa. Key butyrate-producing genera, including *Lachnospira*, *Roseburia*, and *Faecalibacterium*, were significantly diminished, reducing SCFAs synthesis and compromising energy supply to the intestinal epithelium along with barrier integrity.

The observed microbial remodeling likely drives intestinal mucosal inflammation via the “gut microbiota-LPS-TLR4” axis. TAI induced expansion of *Proteobacteria* coincided with elevated LPS, a key component of Gram-negative bacterial membranes. Excessive LPS translocated across the compromised intestinal barrier into the lamina propria, where it bound to TLR4. This interaction upregulated the MyD88 adaptor protein, leading to activation of the canonical NF-κB inflammatory pathway (Mishra et al., 2023; Moresco et al., 2011). This mechanism aligns with recent evidence showing NF-κB hyperactivation as a core driver of inflammatory bowel and radiation-induced intestinal disorders (Jiang X. et al., 2025). TAI significantly increased phosphorylation of TLR4/MyD88/NF-κB cascade components and enhanced expression of the pro-inflammatory cytokines TNF-*α* and IL-6. NF-κB-driven inflammatory signaling has also been implicated in tumor cell proliferation and stress-induced inflammation across diverse tissue models (Hu et al., 2017). These events established a pathogenic cycle: “Microbiota dysbiosis → LPS accumulation → TLR4/NF-κB hyperactivation → Inflammatory cytokine storm.” Consequently, inflammatory cell infiltration increased and radiation-induced intestinal injury was exacerbated. Restoring gut microbiota homeostasis should therefore be prioritized early in the disease course.

TAI induced intestinal metabolic disorders, significantly impacting several key pathways including primary bile acid biosynthesis, taurine and hypotaurine metabolism, sphingolipid metabolism, tryptophan metabolism, and lysine degradation. Comparable multi-omics analyses have shown how pharmacological interventions can reshape key metabolic pathways after neural or systemic injury (Hu et al., 2023). Such microbiota metabolite interactions have been observed in other integrated omics studies, demonstrating the tight linkage between microbial community shifts and metabolic remodeling (Cheng et al., 2022). Among these, sphingolipid metabolism and primary bile acid biosynthesis exhibited the most pronounced alterations. Sphingolipid metabolism represents a critical node within the barrier-immune axis (Zakerska-Banaszak et al., 2024; Jyoti and Dey, 2025). Similarly, microbiota-driven disturbances in lipid metabolism, including sphingolipid signaling, have been implicated in metabolic disorders linked to central nervous system function (Zhu et al., 2022). Intestinal epithelial cells autonomously synthesize sphingolipids via the *de novo* pathway (Espinoza and Snider, 2024), while members of the *Bacteroidetes* phylum—such as *Bacteroides*, *Parabacteroides*, and *Prevotella*—along with certain *Chlorobi* species like *Chlorobium*, supply exogenous atypical sphingolipids (Zakerska-Banaszak et al., 2024), illustrating a bidirectional regulatory relationship between the microbiota and sphingolipids. On one hand, microbiota-derived sphingolipids are recognized by host invariant natural killer T cells (iNKT), modulating local immune homeostasis; on the other, dietary sphingolipids reciprocally influence microbiota composition (Kuo and Hla, 2024). In the TAI model, sphingolipid metabolites increased rapidly after irradiation and gradually returned to baseline in later stages. Time-series analysis suggested that this fluctuation may parallel changes in the relative abundance of *Bacteroidetes*, implying that microbial expansion could drive transient sphingolipid accumulation. Integrating transcriptomic and metabolomic analyses has proven valuable for dissecting complex therapeutic mechanisms in neurological and metabolic disorders (Tang et al., 2023). Taurine and its conjugated bile acids, such as taurocholic acid, negatively regulate de novo sphingolipid synthesis by inhibiting serine palmitoyltransferase (SPT) activity (Espinoza and Snider, 2024); synchronized disruption in taurine metabolism was observed, with its recovery phase coinciding with sphingolipid reduction. This indicates that upregulated taurine metabolism may constitute a key negative feedback mechanism promoting the late-phase normalization of sphingolipid levels. Sphingosine-1-phosphate (S1P), a central signaling molecule in sphingolipid metabolism, regulates immune cell recruitment and activation—including T cells and macrophages—via S1P receptors 1 to 5 (S1PR1–5) (Espinoza and Snider, 2024). Experimental results revealed significant changes in S1P levels following irradiation. Moreover, certain sphingolipid metabolites such as ceramide enhance TLR4 signaling pathway activity and synergistically amplify inflammatory responses with LPS (Zhu and Huang, 2024). These findings provide a theoretical basis for developing radioprotective strategies targeting the microbiota–sphingolipid axis.

Bile acids remodel the gut microecology through two complementary pathways: they selectively promote the proliferation of bile acid-metabolizing taxa while suppressing bile acid-sensitive ones, thereby inducing structural rearrangement of the microbial community (Shi et al., 2023; Larabi et al., 2023). In the colon, primary bile acids (PBAs) are converted into secondary bile acids (SBAs) by specific microbiota—including *Bacteroides*, *Clostridium*, and *Enterococcus*—via reactions such as 7α-dehydroxylation, deconjugation, oxidation, and epimerization (Attema and Kuipers, 2025). Metabolomics data revealed that ionizing radiation significantly perturbed the bile acid profile: taurochenodeoxycholic acid increased sharply at 24 h PI, then declined rapidly and returned to baseline by 3 days. Glycochenodeoxycholic acid, undetectable before irradiation, emerged at 24 h with considerable fluctuation and exhibited a recovery trend by 3 days despite remaining disordered. Glycocholic acid became detectable after irradiation and displayed aggravated metabolic disruption between 24 and 3 days, yet its concentration began to recover following a significant elevation at 3 days.

These disturbances indicated a dynamic “initial activation followed by inhibition” within the PBAs biotransformation pathway following acute radiation injury, accompanied by instability in both the production and diversity of SBAs. SBAs exert direct antibacterial effects by embedding their hydrophobic termini into the cell membranes of Gram-positive bacteria, disrupting membrane lipid ordering and inducing membrane lysis along with the dissociation of membrane proteins (Larabi et al., 2023); more significantly, they act as signaling molecules that concurrently activate host nuclear receptors, such as the Farnesoid X Receptor (FXR), and membrane receptors like GPBAR1 (TGR5), thereby indirectly modulating gut microbiota composition and host immunity (Jiang W. et al., 2025). For example, GPBAR1 activation inhibits the NF-κB pathway, reduces the secretion of pro-inflammatory cytokines, and promotes regulatory T cell (Treg) expansion, thus helping to maintain local and systemic immune homeostasis (Urbani et al., 2025; Peterson and Weidenmaier, 2025). Previous studies have confirmed that certain bacteria within the *Firmicutes* phylum—including *Clostridium bolteae*, *C. scindens*, and *Lactobacillus* species—harbor complete bile acid metabolic enzyme systems. These bacteria can promote intestinal epithelial regeneration and mucosal healing via TGR5 signaling (Jalil et al., 2025; Daniel and Ridlon, 2025). This study revealed a significant PI decrease in the relative abundance of *Firmicutes*, along with transient expansion of potentially pathogenic bacteria. Concurrently, PBAs metabolic dysregulation exhibited a significant positive correlation with an aberrant microbial community structure. These findings suggest that a vicious cycle: “gut microbiota dysbiosis → impaired PBAs synthesis/conversion → weakened SBAs signaling → further gut microbiota dysbiosis”—may underlie the disruption of microecological-immune interactions after acute radiation injury. Consequently, targeted restoration of the bile acid metabolic pathway, especially by enhancing the efficient conversion of PBA to SBAs, could offer a viable strategy for rebuilding gut microbiota and repairing intestinal mucosal barriers following acute radiation exposure.

Amino acids serve not only as fundamental building blocks of proteins but also as key precursors in metabolic and immune signaling networks. They act as substrates for signaling molecules such as SCFAs, serotonin, and *γ*-aminobutyric acid (GABA), directly contributing to host–microbe co-metabolism; meanwhile, gut amino acid homeostasis is dynamically maintained by the microbiota through decomposition, resynthesis, and transformation processes, thereby regulating host energy metabolism and immune homeostasis (Miao et al., 2025; Lin et al., 2017). Lysine, for instance, is metabolized by specific bacterial communities in the small and colonic lumina. Through the lysine → butyryl-CoA → butyrate pathway, it contributes to the butyrate pool alongside threonine, glutamate, and other precursors (Wang et al., 2022; Dominiak et al., 2025). Butyrate activates the G protein-coupled receptor GPR43 (Free Fatty Acid Receptor 2, FFAR2), which upregulates the AMPK/PGC-1*α* axis to boost energy production in colonic epithelial cells while suppressing NF-κB signaling and reducing pro-inflammatory cytokine release (TNF-α, IL-6). This in turn enhances the expression of tight junction proteins such as claudin-1 and occludin, thereby reinforcing intestinal barrier integrity (Dominiak et al., 2025). Findings reveal pronounced stress-induced fluctuations in lysine metabolism: degradation intensified markedly at 24 h post-irradiation, suggesting mobilization of the lysine–butyrate axis for acute barrier repair, while by 3 days, lysine levels trended toward recovery, likely due to microbial resynthesis from ammonia-nitrogen (Smythe and Efthimiou, 2022). Tryptophan metabolism was also dysregulated, with tryptophan-indole pathways modulated by commensals such as Clostridium and Lactobacillus via cross-feeding, leading to significantly altered luminal amino acid profiles and resynthesis of multiple essential amino acids (Miao et al., 2025; Zheng et al., 2025). These metabolic shifts correlate with reduced microbial alpha diversity and elevated energy expenditure, further underscoring the centrality of the microbiota–amino acid–energy metabolism axis in irradiation stress.

Mirroring disturbances in the amino acid–microbiota axis, the overall content of SCFAs decreased significantly after irradiation, with a sharp reduction at 24 h and only partial recovery by 3 days, leaving levels persistently suboptimal. SCFAs are primarily generated through bacterial fermentation of dietary fiber or amino acids by members of the *Bacteroidetes* and *Firmicutes* phyla, as well as lactic acid bacteria. Botanical supplements such as *Artemisia argyi* have been demonstrated to promote intestinal epithelial repair and improve barrier functions via antioxidant and microbial regulatory effects (Chen et al., 2022). These metabolites mediate hormone secretion, epigenetic modifications, and immune regulation via receptors such as FFAR2/3 and GPR109A (Cheng et al., 2024; Zheng et al., 2025). SCFAs inhibit the NF-κB signaling pathway, attenuate pro-inflammatory macrophage polarization, and promote the differentiation of anti-inflammatory M2-type macrophages; they also induce expression of tight junction proteins, stimulate MUC2 (Mucin 2) mucus secretion, and remodel the gut microbiota structure. Furthermore, SCFAs supply energy to the intestinal epithelium and symbiotic bacteria, thereby reinforcing barrier integrity (Bernardi et al., 2024). Consequently, irradiation-induced SCFAs depletion not only compromised GPR43-mediated energy supply to enterocytes—where butyrate satisfies approximately 70% of colonocyte energy demand (Cheng et al., 2024)—but also promoted LPS translocation and activation of inflammatory signaling pathways. This initiated a vicious cycle of dysregulation within the “Amino Acid–Gut Microbiota–SCFAs–Barrier” axis, further aggravating intestinal injury.

## 5 Conclusion

In summary, TAI systematically disrupted the intestinal epithelial barrier, causing intestinal tissue damage and inducing gut dysbiosis marked by a reduced relative abundance of *Firmicutes* and the proliferation of *Proteobacteria*, *Shigella*, and other harmful bacteria. Elevated gut microbiota-derived LPS activated the TLR4/MyD88/NF-κB pathway, triggering inflammatory responses mediated by TNF-*α* and IL-6. Gut microbiota dysbiosis, along with disruptions in bile acid and sphingolipid metabolic pathways and the depletion of SCFAs and essential amino acids, collectively promoted metabolic disorders. These alterations further impaired intestinal barrier repair and exacerbated intestinal damage, establishing a vicious cycle of “intestinal injury—gut dysbiosis—metabolic disorder—inflammatory response.”

Prompt prevention and treatment of acute intestinal injury induced by TAI may be a potentially effective measure to alleviate radiation-induced intestinal injury. Based on previous literature, we may consider establishing a comprehensive intervention system around the following four aspects. First, glutamine, epidermal growth factor, or SCFAs such as butyrate could be administered around the time of irradiation to strengthen tight junction protein expression and support epithelial repair. Second, to counter gut microbiota dysbiosis, composite probiotics dominated by *Firmicutes* (e.g., *Faecalibacterium* and *Roseburia*) and butyrate-producing *Clostridium* strains may be introduced, along with prebiotics like fructooligosaccharides and resistant starch, to inhibit the expansion of harmful bacteria such as *Proteobacteria* and *Shigella*. Concurrently, broad-spectrum antibiotics should be used restrictively. Third, metabolic regulation might involve a diet enriched with SBAs to correct bile acid metabolic disorders, alongside supplementation with essential amino acids—including tryptophan, lysine, and threonine—to boost SCFAs synthesis and preserve intestinal barrier function. Finally, for inflammation control, prophylactic use of TLR4 inhibitors or NF-κB pathway blockers, combined with antioxidant nutrients (vitamins C and E) and *ω*-3 polyunsaturated fatty acids, could mitigate LPS-induced activation of the TLR4/MyD88/NF-κB pathway and reduce secretion of pro-inflammatory cytokines like TNF-α and IL-6. These strategies are designed to work synergistically, forming an integrated protective framework described as “barrier reinforcement—gut microbiota remodeling—metabolic correction—inflammation suppression,” thereby maximizing mitigation of radiation-induced intestinal damage.

This study offers a comprehensive analysis of intestinal tissue damage, gut microbiota, metabolome, and inflammatory signaling during acute radiation injury, but its short observation period limits understanding of long-term effects and complications. Future work should extend into the subacute and chronic phases after radiation to monitor long-term dynamics. Integrating multi-omics with clinical assessments and testing interventions such as probiotics would further enhance its value. Our short-term results establish a foundation for extended studies, which are essential for comprehensively elucidating radiation-induced processes and improving long-term patient management.

## Data Availability

The datasets presented in this study can be found in online repositories. The names of the repository/repositories and accession number(s) can be found below: NCBI (accession: PRJNA1348346).
